# Endothelial autophagy and Endothelial-to-Mesenchymal Transition (EndoMT) in eEPC treatment of ischemic AKI

**DOI:** 10.1007/s40620-015-0222-0

**Published:** 2015-08-20

**Authors:** Daniel Patschan, Katrin Schwarze, Elvira Henze, Susann Patschan, Gerhard Anton Müller

**Affiliations:** Clinic of Nephrology and Rheumatology, University Medical Center of Göttingen, Robert-Koch-Straße 40, 37075 Göttingen, Germany

**Keywords:** AKI, Endothelial autophagy, EPCs, EndoMT

## Abstract

**Background:**

Autophagy enables cells to digest endogenous/exogenous waste products, thus potentially prolonging the cellular lifespan. Early endothelial progenitor cells (eEPCs) protect mice from ischemic acute kidney injury (AKI). The mid-term prognosis in AKI critically depends on vascular rarefication and interstitial fibrosis with the latter partly being induced by mesenchymal transdifferentiation of endothelial cells (EndoMT). This study aimed to determine the impact of eEPC preconditioning with different autophagy inducing agents [suberoylanilide hydroxamic acid (SAHA)/temsirolimus] in ischemic AKI.

**Methods:**

Male C57/Bl6 N mice were subjected to bilateral renal ischemia (40 min). Animals were injected with either untreated, or SAHA- or temsirolimus-pretreated syngeneic murine eEPCs at the time of reperfusion. Mice were analyzed 48 h and 4 weeks later. In addition, cultured eEPCs were treated with transforming growth factor (TGF)-β ± SAHA, autophagy (perinuclear LC3-II), and stress-induced premature senescence (SIPS—senescence-associated β-galactosidase, SA-β-Gal), and were evaluated 96 h later.

**Results:**

Cultured eEPCs showed reduced perinuclear density of LC3-II + vesicles and elevated levels of SA-β-Gal after treatment with TGF-β alone, indicating impaired autophagy and aggravated SIPS. These effects were completely abrogated by SAHA. Systemic administration of either SAHA or tems pretreated eEPCs resulted in elevated intrarenal endothelial p62 at 48 h and 4 weeks, indicating stimulated endothelial autophagy. This effect was most pronounced after injection of SAHA-treated eEPCs. At 4 weeks endothelial expression of mesenchymal alpha-smooth muscle actin (αSMA) was reduced in animals receiving untreated and SAHA-pretreated cells. In addition, SAHA-treated cells reduced fibrosis at week 4. Tems in contrast aggravated EndoMT. Postischemic renal function declined after renal ischemia and remained unaffected in all experimental cell treatment groups.

**Conclusion:**

In ischemic AKI, intrarenal endothelial autophagy may be stabilized by systemic administration of pharmacologically preconditioned eEPCs. Early EPCs can reduce postischemic EndoMT and fibrosis in the mid-term. Autophagy induction in eEPCs either increases or decreases the mesenchymal properties of intrarenal endothelial cells, depending on the substance being used. Thus, endothelial autophagy induction in ischemic AKI, mediated by eEPCs is not a renoprotective event per se.

## Introduction

Early endothelial progenitor (or outgrowth) cells (eEPCs/eEOCs) have been proven as an effective therapeutic tool in murine ischemic acute kidney injury (AKI) [1, 2]. As opposed to so-called *late* EPCs (lEPCs), early EPCs predominantly act by indirect mechanisms (e.g., modulation of the perivascular milieu by producing proangiogenic substances and/or by releasing vasoprotective microparticles, enriched by certain micro-RNA molecules). In the current literature, lEPCs are defined as true *progenitors* of endothelial cells whereas eEPCs are classified as ‘hematopoietic cells with proangiogenic activity’. Nevertheless, early EPCs have successfully been used to treat ischemic diseases under diverse experimental conditions (e.g., ischemic heart and cerebrovascular disease). In 2006, the cells were shown to promote post-AKI repair as well. Meanwhile, a significant number of pharmacological strategies have been established, helpful to increase renoprotective competence of eEPCs in ischemic AKI [3–7]. The microenvironmental alterations associated with ischemia can significantly aggravate renal damage since ischemia does not only induce tubular malfunction/damage but also interstitial inflammation and severe microvasculopathy [8]. The latter causes ongoing ischemia even if the initial cause of hypoperfusion has been eliminated. Both postischemic interstitial inflammation and microvasculopathy significantly result from alterations of the paracrinic milieu. The proximal tubule releases numerous factors including tumor necrosis factor (TNF)-α, interleukin (IL)-6, IL-1β, TGF-β, monocyte chemoattractant protein-1 [MCP-1], IL-8, and RANTES [9–11]. In such a context, mature endothelial cells undergo aggravated senescence or stress-induced premature senescence (SIPS) [12]. eEPCs, after invading the kidney via the renal artery, are most likely also altered by deleterious effects of diverse humoral factors and it can be argued that SIPS decreases the anti-ischemic competence of eEPCs in AKI. A few years ago, studies by the group of Goligorsky elegantly showed a dynamic cascade of hyperglycemia-induced inhibition of endothelial autophagy, followed by increased endothelial SIPS [13]. Autophagy is widely regarded as an endogenous defense mechanism against numerous endogenous and exogenous stressors. The role of autophagy in modulating the ‘renal injury-response’ has especially been analyzed in the tubular compartment. Periyasamy-Thandavan and colleagues identified autophagy as a cytoprotective mechanism in cisplatin-induced damage of proximal tubular epithelial cells [14]. Another study showed autophagy to have a protective role during in vitro hypoxia and in vivo ischemia–reperfusion injury (IRI) [15]. Finally, Quercetin-mediated attenuation of renal IRI was shown to critically depend on autophagy activation in an AMP-activated protein kinase-dependent manner [16]. Therefore one may conclude that stimulated (tubular) autophagy generally increases tissue resistance against toxic/ischemic damage. Nevertheless, de facto no study has so far evaluated the role of endothelial autophagy in AKI. Thus, the aim of our study was to analyze whether stabilization of autophagy in therapeutically administered eEPCs increases renoprotective cell competence in murine ischemic AKI.

## Methods

### Animal models

The animal study protocol was in accordance with the guidelines of the German Institute of Health Guide for the Care and Use of Laboratory Animals and ap-proved by the Institutional Animal Care and Use Committee. C57BL/6 N mice were originally obtained from Jackson Labs (Bar Harbor, ME, USA) and bred in the local animal facility of the Göttingen University Hospital. As in previous studies, male 8-12 week-old C57Bl/6 N mice were used in all experiments. All animals were separately caged with a 12:12-h light–dark cycle and had free access to water and chow throughout the study.

### Surgical procedures

Mice were anesthetized (300 μl of 6 mg/100 g ketamine hydrochloride plus 0.77 mg/100 g xylazine hydrochloride) and placed on a heated surgical pad. Rectal temperature was maintained at 37 °C. After a 1.5-cm mid-laparotomy, the right kidney was removed. The left kidney was exposed and clamping of the renal pedicle was performed with microserrefines (Fine Science Tools, Foster City, CA, USA). For induction of AKI, we performed unilateral renal artery clamping post-uninephrectomy. The respective procedure has been used in several earlier studies [4, 17, 18]. Briefly, first, a suture was placed around the right renal artery, vein, and ureter, respectively. The suture was held open until cell injection. Simultaneously, the left renal pedicle was transiently (40 min) occluded. At the end of the ischemic period a certain volume of eEPC-containing EBM-2 media (0.5 × 10^6^ eEOCs in 100 µl) was injected into the right renal vein (systemic circulation). Very shortly after cell injection, the suture was closed in order to prevent bleeding. The kidney was removed afterwards. AKI resulted from transient occlusion of the left renal pedicle, followed by contralateral nephrectomy. The abdominal incision was closed with a 4–0 suture and surgical staples. In each experimental group 10 animals were analyzed. Animals were sacrificed at hour 48 or at week 4 postischemia. The experimental conditions were chosen since earlier studies showed that AKI cannot be prevented if 0.5 × 10^6^ eEPCs are administered after acute renal ischemia of 40 min. Thus, these were optimal conditions to test the hypothesis that endothelial autophagy stabilization may improve postischemic kidney function.

### Culture of mouse-derived early endothelial progenitor cells

In order to perform cell injection experiments eEPCs were isolated from C57Bl/6 N mice as described previously [17, 19]. Briefly, mouse mononuclear cells (MNCs) were enriched by density gradient centrifugation using Biocoll solution (Biochrom, Berlin, Germany) from peripheral blood and spleen cell extracts. The reason for pooling MNCs was to maximize the total number of cells available for injection. Immediately following isolation, MNCs were mixed and 4 × 10^6^ cells were plated on 24-well culture dishes coated with human fibronectin (Sigma, St Louis, MO, USA) and maintained in endothelial cell growth medium-2 (EGM-2 - Clonetics, Lonza, Walkersville, MD, USA) supplemented with EGM single-quots containing 5 % fetal calf serum (FCS). After 4–5 days of culture, eEPCs were identified by the uptake of DiI-labeled acetylated low density lipoprotein (acLDL) (Invitrogen, Carlsbad, CA, USA) and binding of FITC-labeled BS-1 lectin (BS-1) (Sigma Diagnostics, St. Louis, MO, USA). For in vitro cell experiments, a murine endothelial progenitor cell line was purchased (66110-37—Celprogen stem cell research and therapeutics, San Pedro, CA, USA) and cultured according to the manufacturer’s protocol. Pharmacological preconditioning was performed using either suberoylanilide hydroxamic acid (SAHA) or temsirolimus at the following concentrations: SAHA (5 µmol), temsirolimus (10 µg/ml). Cell incubation was performed in EGM for 1 h at 37 °C. After washing the cells with EGM, 0.5 × 10^6^ eEPCs were resuspended in 100 μl EGM for systemic injection.

### Cell culture experiments

Primary murine endothelial progenitor cells (MEPC—Celprogen, 66110-37) were incubated with either SAHA alone (5 µmol) or with SAHA and TGF-β (5 ng/ml) combined for 1 h at 37 °C. Treatments were performed in EGM. After washing the cells, LC3-II was stained with anti-LC3 rabbit (Cell Signaling, 2775), followed by secondary incubation with anti-rabbit IgG-NL 493 (NL006, R&D Systems, Minneapolis, MN, USA). Primary incubation was performed overnight at 4 °C while secondary incubation was performed for 1 h at room temperature. Staining of senescence-associated beta-galactosidase (SA-β-Gal) was performed using a commercially available kit (Signaling, 9860) according to the manufacturer´s protocol. Autophagy was quantified by measuring the density of LC3^+^ perinuclear vesicles using ImageJ software. SIPS was quantified by measuring the cell surface area covered by blue staining signals (%) (ImageJ).

### Histology including immunofluorescence of tissue sections

For quantification of kidney fibrosis, formalin-fixated, paraffin-embedded tissue sections were stained with Masson Trichrome. The amount of collagen deposition was assed using ImageJ software. Fibrosis was given as the mean percentage of three view fields covered by the color green. For evaluating EndoMT, formalin-fixated, paraffin-embedded tissue sections were stained with rat anti-mouse CD31 (PECAM-1—CloneSZ31, Dianova) and rabbit anti-SMA (EMELCA) for primary incubation and with Alexa Fluor 488 goat anti-rabbit IgG (Dianova) and Alexa Fluor 594 goat anti-rat IgG (Dianova) for secondary incubation. Primary incubation was performed overnight at 4 °C while secondary incubation was performed for 1 h at room temperature. In accordance with a more recent publication about methodological approaches for autophagy visualization, p62 and not LC3-II was used for detecting autophagocytic activity in tissue sections [20]. Staining of p62 was performed with rabbit anti p62 (abcam ab91526) for primary incubation (4 °C, overnight), followed by secondary incubation with anti-rabbit 488 (Jackson ImmunoResearch, West Grove, PA, USA) for 1 h at RT. To visualize the nuclei, tissue sections were counterstained with DAPI. Three view fields per kidney were analyzed for colocalization of either αSMA or p62 and CD31 using ImageJ software.

### Analysis of renal function

Serum creatinine concentration was measured using a commercially available kit (Creatinin, Jaffé, Labor und Technik, Eberhard Lehmann, LT-CR0121, Berlin, Germany) according to the manufacturer’s protocol.

### Statistical analysis

The results were expressed as mean ± standard error of the mean (SEM). The means of two populations were compared by Student’s *t* test or Mann–Whitney U test. Differences were considered significant at p < 0.05.

## Results

### Pharmacological autophagy activation reduces SIPS in cultured early endothelial progenitor cells

**Fig. 1:**
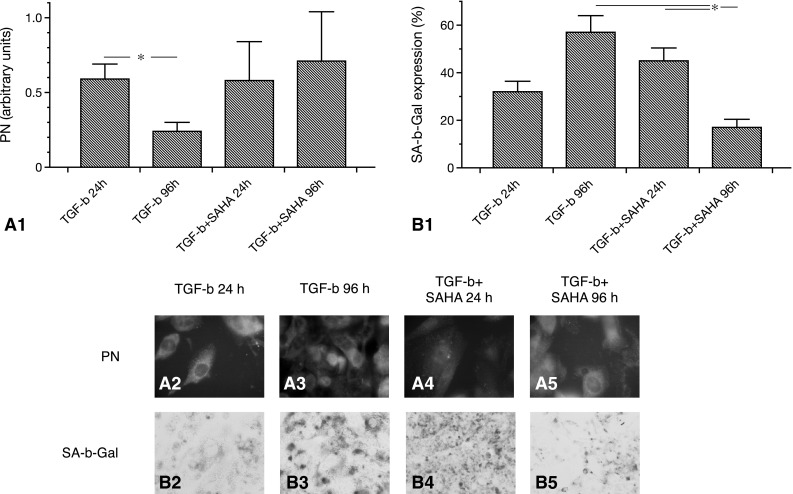
Autophagy and SIPS in cultured murine eEPCs exposed to TGF-β with vs. without SAHA treatment. **a.1** Shows time-courses of cellular autophagy, as reflected by the perinuclear density (PN) of LC3-II^+^ vesicles. TGF-β alone decreased PN from hour 24 to 96, SAHA completely stabilized PN. **a.2**–**a.5** Representative images of LC3-II stained cells with vs. without SAHA treatment (magnification ×100). **b.1** Displays dynamics of cellular SIPS with decreased SA-β-Gal positivity at hour 96 after combined incubation with TGF-β and SAHA. Representative images of SA-β-Gal stained cells are shown in images **b.2**–**b.5** (magnification ×40) (data as mean ± SEM, *p < 0.05). eEPCs, early endothelial progenitor cells; TGF, transforming growth factor; SAHA, suberoylanilide hydroxamic acid; SIPS, stress-induced premature senescence

The first experiments were related to the dynamics of autophagy and SIPS in cultured murine eEPCs. In order to induce SIPS, cells were treated with TGF-β. SIPS was evaluated by staining the cells for SA-β-Gal (% of cell surface area stained positive). TGF-β treatment resulted in increased SA-β-Gal positivity after 96 as compared to 24 h: 96 h 57.4 ± 7.1 % vs. 24 h 31.4 ± 4.4 %. The difference was close to the level of significance (p = 0.06). Next, cells were coincubated with TGF-β and the autophagy inducing substance SAHA. At 24 h, SA-β-Gal positivity was 45.8 ± 5.4 %. The difference between SAHA and non-SAHA treated cells was not significant at this time (p = 0.18). At 96 h, in contrast, SA-β-Gal was markedly reduced as compared to 24 h (+SAHA) and as compared to 96 h without SAHA coincubation: 96 h +SAHA 17.3 ± 3.4 % vs. 24 h +SAHA 45.8 ± 5.4 % and vs. 96 h −SAHA 57.4 ± 7.1 %, p = 0.02 and p = 0.02) (Fig. 1). In order to analyze autophagy, cells were stained for LC3-II. Autophagocytic activity was quantified by measuring the density of perinuclear LC3-II^+^ vesicles (perinuclear granularity, PN, arbitrary units). Without SAHA, TGF-β induced a significant PN decrease from hour 24 to hour 96: 24 h 0.6 ± 0.1 vs. 96 h 0.24 ± 0.07, p = 0.03. SAHA coincubation in contrast stabilized PN from hour 24 to 96: 24 h +SAHA 0.58 ± 0.26 vs. 96 h +SAHA 0.71 ± 0.33, p = 0.77 (Fig. 1).

### Preconditioned eEPCs stimulate autophagy in intrarenal endothelial cells

**Fig. 2:**
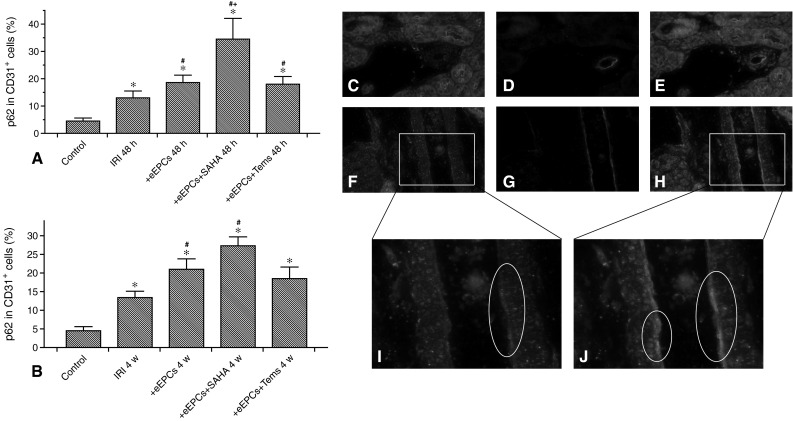
Intrarenal endothelial autophagy after IRI with versus without systemic administration of unconditioned or preconditioned eEPCs. **a** Percentages of endothelial p62 in the short-term (48 h). Endothelial presence of p62 increased after IRI in all experimental groups. Administration of unconditioned and preconditioned eEPCs further elevated p62 with a peak in the ‘eEPC + SAHA’-group. **b** Endothelial p62 at week 4 after IRI. **c**–**j** p62 (*green*) in the endothelium of a small artery (CD31—*red*; **c**–e control, **f**–**h** post-IRI at 48 h). Images **i** and **j** magnify the endothelial layer. The white ovals in **i** and **j** surround the endothelium in areas of increased p62 presence (Tems: temsirolimus; magnification in C–H ×20, in I and J ≈ ×40; Data in A and B are mean ± SEM, ‘*’ indicates significant differences (p < 0.05) as compared to untreated controls, ‘#’ indicates significant differences as compared to the ‘IRI’-group, ‘+’ indicates the difference between ‘+eEOCs’ and ‘+ eEOCs + SAHA’—for exact p-values see text). IRI, ischemia–reperfusion injury; eEOCs, early endothelial outgrowth cells. For other abbreviations, see previous figure (color figure online)

Next, we aimed to analyze whether systemic administration of preconditioned eEPCs to postischemic mice would modulate autophagy in mature endothelial cells of smaller arteries/arterioles. Cell preconditioning was performed using two different autophagy inducing agents, the substance SAHA and the mammalian target of rapamycin (mTOR) inhibitor temsirolimus. Endothelial autophagy was measured by staining of p62 in CD31^+^ cells, and analyzed at 48 h and 4 weeks postischemia. The results shall be given separately for 48 h and 4 weeks. At 48 h (p62 in CD31^+^ cells in  %): Control 4.5 ± 1.1 %, IRI 13.1 ± 2.5 %, IRI+eEPCs 18.6 ± 2.7 %, IRI+eEPCs+SAHA 34.5 ± 7.6 %, IRI+eEPCs+temsirolimus 18 ± 2.8 %. The following differences were significant: Control vs. IRI (p = 0.0051) vs. IRI+eEPCs (p = 0.001) vs. IRI+eEPCs+SAHA (p < 0.0001) vs. IRI+eEPCs+temsirolimus (p < 0.0001); IRI vs. IRI+eEPCs (p = 0.028) vs. IRI+eEPCs+SAHA (p = 0.0032) vs. IRI+eEPCs+temsirolimus (p = 0.047); IRI+eEPCs vs. IRI+eEPCs+SAHA (p = 0.033). At 4 weeks: IRI 13.4 ± 1.7 %, IRI+eEPCs 21 ± 2.3 %, IRI+eEPCs+SAHA 27.3 ± 2.4 %, IRI+eEPCs+temsirolimus 18.5 ± 3.2 %. The following differences were significant: Control vs. IRI (p = 0.0017) vs. IRI+eEPCs (p = 0.0003) vs. IRI+eEPCs+SAHA (p < 0.0001) vs. IRI+eEPCs+temsirolimus (p = 0.00017); IRI vs. IRI+eEPCs (p = 0.023) vs. IRI+eEPCs+SAHA (p = 0.0029) (Fig. 2).

### Preconditioned eEPCs fail to improve postischemic kidney function in the short- and mid-term

**Fig. 3:**
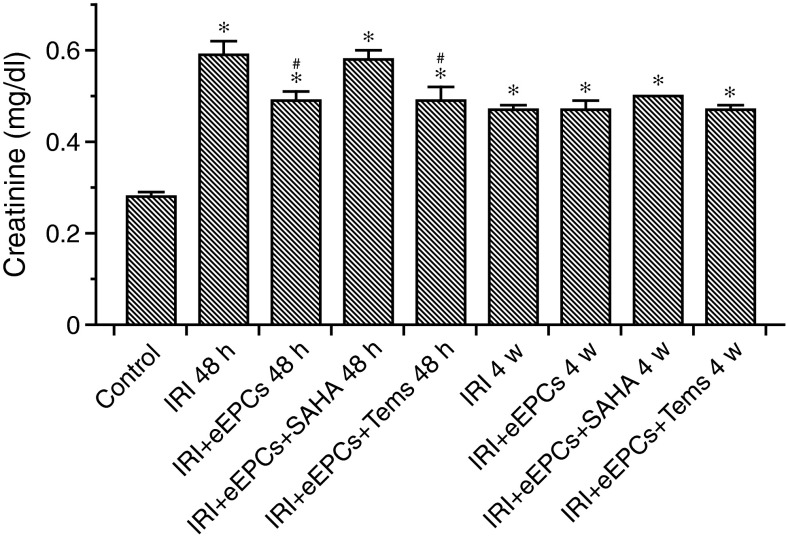
Postischemic kidney function in all experimental groups. Ischemia diminished excretory function until week four. Cell therapy did not improve serum creatinine levels with one exception (native eEPCs at 48 h) (Data are mean ± SEM, ‘*’ indicates significant differences (p < 0.05) as compared to untreated controls, ‘#’ indicates differences as compared to the ‘IRI’-group–for exact p-values, see text)

Excretory kidney function was significantly affected in all postischemic mice. At 48 h (serum creatinine in mg/dl): Control 0.28 ± 0.01, IRI 0.59 ± 0.03, IRI+eEPCs 0.49 ± 0.02, IRI+eEPCs+SAHA 0.58 ± 0.02, IRI+eEPCs+temsirolimus 0.49 ± 0.03. At 4 weeks: IRI 0.47 ± 0.02, IRI+eEPCs 0.47 ± 0.02, IRI+eEPCs+SAHA 0.5 ± 0, IRI+eEPCs+temsirolimus 0.47 ± 0.01. The p-values were: Control vs. IRI 48 h (p < 0.0001) vs. IRI+eEPCs 48 h (p < 0.00t01) vs. IRI+eEPCs+SAHA 48 h (p < 0.0001) vs. IRI+eEPCs+temsirolimus 48 h (p = 0.00053) vs. IRI 4 weeks (p < 0.0001) vs. IRI+eEPCs 4 weeks (p < 0.0001) vs. IRI+eEPCs+SAHA 4 weeks (p < 0.0001) vs. IRI+eEPCs+temsirolimus 4 weeks (p < 0.0053). Finally, administration of unconditioned and temsirolimus pretreated eEPCs improved kidney function at 48 h: IRI vs. IRI+eEPCs (p = 0.019) vs. IRI+eEPCs+temsirolimus (p = 0.037) (Fig. 3).

### Native and preconditioned eEPCs mediate antimesenchymal effects in the mid-term

**Fig. 4:**
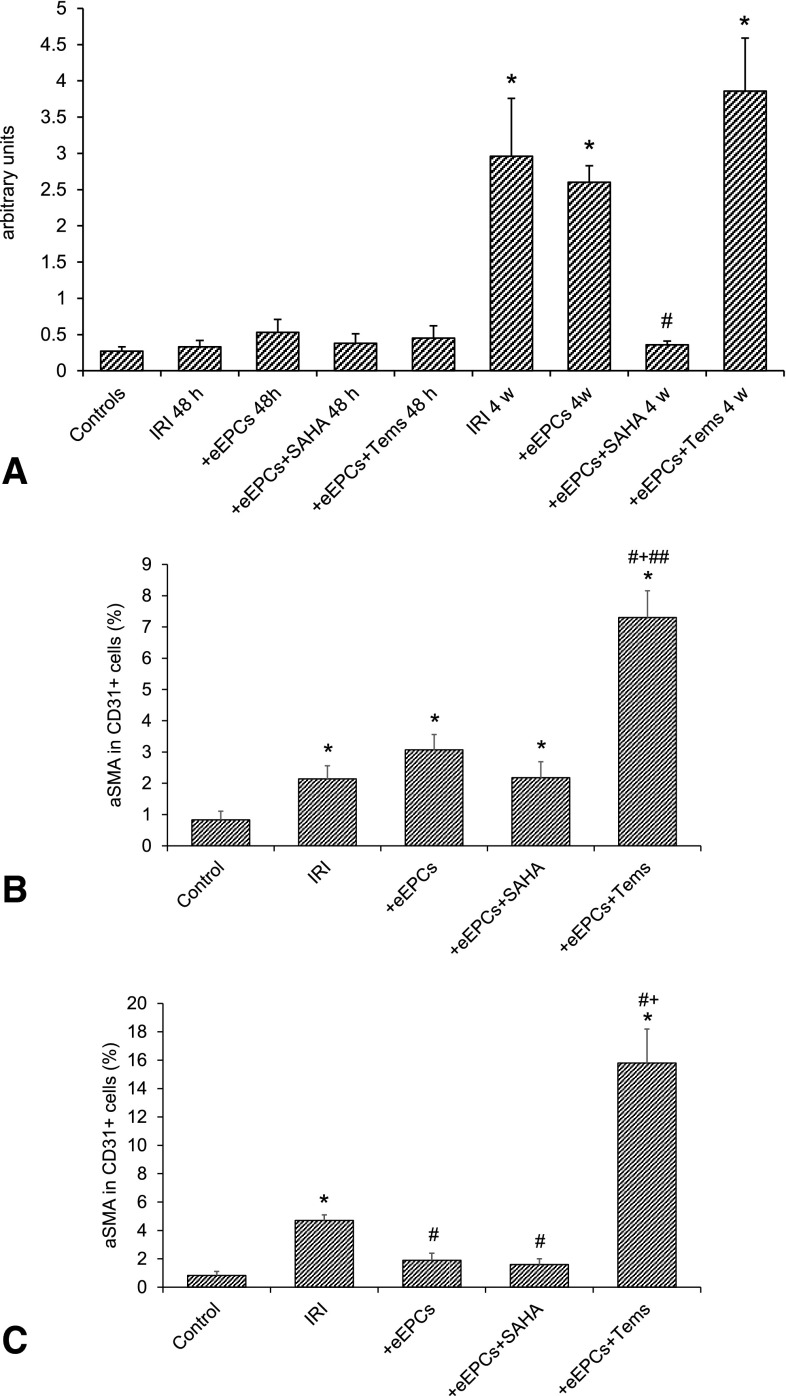
Renal fibrosis and EndoMT in the respective groups. Significant fibrosis exclusively occurred after 4 weeks but not at 48 h postischemia (**a**). Administration of SAHA treated cells mediated anti-mesenchymal effects at week 4. **b** EndoMT at 48 h postischemia. Endothelial cells within small arteries/arterioles displayed increased expression of alpha-smooth muscle actin in all experimental groups, the strongest pro-mesenchymal transformation was observed in animals receiving temsirolimus-treated eEPCs. **c** EndoMT at week 4 postischemia. While temsirolimus-treated cells further aggravated EndoMT, administration of native and SAHA preconditioned eEPCs stabilized endothelial properties of the cells [data as mean ± SEM, ‘*’ indicates significant differences (p < 0.05) as compared to untreated controls, ‘#’ in **a** difference between ‘IRI’ and ‘+eEPCs+SAHA 4 w’; ‘#’ in **b** difference between ‘IRI’ and ‘+eEPCs+Tems’; ‘#’ in **c** differences as compared to ‘IRI’; ‘+’ in **b** difference between ‘+eEPCs’ and ‘+eEPCs+Tems’; ‘+’ in **c** difference between ‘IRI’ and ‘+eEPCs+Tems’; ‘##’ in **b** difference between ‘+eEPCs+SAHA’ and ‘+eEPCs+Tems’–for exact p-values see text]. EndoMT, mesenchymal transdifferentiation of endothelial cells. For other abbreviations, see previous figures

Endothelial-to-mesenchymal transition significantly contributes to tissue fibrosis in the kidney and in other organs such as the heart [19, 21, 22]. Fibrosis was analyzed by Masson trichrome staining, the results being expressed in relative units. At 48 h: Control 0.27 ± 0.06, IRI 0.33 ± 0.09, IRI+eEPCs 0.53 ± 0.18, IRI+eEPCs+SAHA 0.38 ± 0.13, IRI+eEPCs+temsirolimus 0.45 ± 0.17. The differences were not statistically significant at 48 h. Four weeks: IRI 2.9 ± 0.8, IRI+eEPCs 2.6 ± 0.23, IRI+eEPCs+SAHA 0.36 ± 0.05, IRI+eEPCs+temsirolimus 3.86 ± 0.73. The following differences were significant: Control vs. IRI (p < 0.0001) vs. IRI+eEPCs (p < 0.0001) vs. IRI+eEPCs+temsirolimus (p < 0.0001). Endothelial-to-mesenchymal transition was evaluated by tissue staining of αSMA and Cat D31. The results are given separately for 48 h and 4 weeks. At 48 h (αSMA in CD31^+^ cells in  %): Control 0.8 ± 0.28 %, IRI 2.14 ± 0.42 %, IRI+eEPCs 3.07 ± 0.49 %, IRI+eEPCs+SAHA 2.18 ± 0.51 %, IRI+eEPCs+temsirolimus 7.3 ± 0.86 %. The following differences were significant: Control vs. IRI (p = 0.017) vs. IRI+eEPCs (p = 0.0025) vs. IRI+eEPCs+SAHA (p = 0.0075) vs. IRI+eEPCs+temsirolimus (p < 0.0001); IRI+eEPCs+temsirolimus vs. IRI (p < 0.0001) vs. IRI+eEPCs (p = 0.003) vs. IRI+eEPCs+SAHA (p < 0.0001). At 4 weeks: IRI 4.7 ± 0.4 %, IRI+eEPCs 1.8 ± 0.6 %, IRI+eEPCs+SAHA 1.65 ± 0.4 %, IRI+eEPCs+temsirolimus 15.8 ± 2.5 %. The following differences were significant: Control vs. IRI (p < 0.0001) vs. IRI+eEPCs+temsirolimus (p < 0.0001); IRI vs. IRI+eEPCs (p = 0.0037) vs. IRI+eEPCs+SAHA (p = 0.005); IRI+eEPCs+temsirolimus vs. IRI (p < 0.0001) vs. IRI+eEPCs (p = 0.0014) vs. IRI+eEPCs+SAHA (p < 0.0001) (Fig. 4).

## Discussion

In this study we report consequences of endothelial autophagy (AP) stabilization in ischemic AKI for the first time. In many situations autophagy acts as an endogenous defense mechanism against numerous stressors of exogenous and endogenous origin [20]. Autophagy activation can prolong the cellular lifespan, depending on the microenvironmental circumstances that cells are being exposed to. The role of AP in AKI has been investigated by several investigators in the past. However, no study ever evaluated the particular role of endothelial autophagy. Tubular AP, in contrast, has been the object of interest in recent years. Chen and colleagues showed attenuation of renal ischemia/reperfusion injury via activation of the AMP-activated protein kinase-regulated autophagy pathway [16]. Another study identified autophagy as a cytoprotective mechanism during cisplatin injury of renal proximal tubular cells [14]. Finally, Howell and colleagues showed autophagy augmentation as an effective approach to treat acute kidney injury during endotoxemia in mice [23]. Together, these investigations point towards a protective role of (tubular) AP in AKI. However, a more recent and yet unpublished study by Schmitt and colleagues (ASN meeting 2014) showed that AP, although protecting the tubular compartment in the short term, may aggravate the risk for SIPS in the mid- to long-term, thus increasing the risk for chronic kidney disease (CKD).

Our data clearly indicate that eEPC autophagy can be stimulated by pharmacological measures. TGFβ-induced SIPS on the other hand is diminished. Systemic administration of AP-stimulated eEPCs activates the autophagocytotic cascade in intrarenal endothelial cells (small arteries/arterioles). Nevertheless, induced endothelial AP is not associated to any improvement of postischemic renal function either in the short- or in the mid-term. However, both, native and SAHA-preconditioned eEPCs inhibit EndoMT in the mid-term and SAHA-treated cells diminish fibrosis at this time as well. The general conclusions that may be drawn from the study are: (1) endothelial autophagy activation (via eEPCs) is not a renoprotective measure per se. The (pharmacological) protocol being for used for AP induction is fundamentally important; (2) although induced endothelial AP is associated with reduced EndoMT under certain circumstances, any mechanistic relationship between the two processes cannot be proposed at the moment; (3) the autophagy inducer SAHA enables eEPCs to act as an antifibrotic. These effects are not exclusively mediated by modulation of EndoMT since native eEPCs reduced EndoMT as well but fibrosis did not differ from that in animals receiving SAHA-preconditioned cells. Organofibrogenesis is a complex process ultimately resulting in tissue scaring and dysfunction [24]. Although activated fibroblasts have been proposed as the major source of collagen/matrix production, further cell types have been identified to differentiate into pro-fibrotic cells under certain circumstances. Among those are endothelial and smooth-muscle cells, and pericytes [24]. Finally, tubular epithelial cells in the kidney may acquire a mesenchymal phenotype (EMT—epithelial-to-mesenchymal transition) [25]. Early EPCs modulate the postischemic microenvironment by secreting a multitude of humoral factors and by releasing vasomodulatory microparticles [26–29]. The latter have been shown to reduce tubular cell apoptosis as well [27] and possible long-term effects on mesenchymal properties of tubular (and other types of) cells may be presumed. Most studies performed in the past failed to show significant incorporation of eEPCs into the endothelial layer of small blood vessels, which is in line with the concept of indirect cell activity within the perivascular compartment. In the current study we avoided cell tracing using an exogenous dye. In an older study *Celtracker*^®^-labelled eEPCs significantly deteriorated postischemic kidney function after being preconditioned with angiopoietin-1. Thus, exogenous tracing strategies may potentially be more harmful than beneficial.

In summary, a renoprotective role for endothelial AP in AKI cannot definitely be proposed at the moment. Endothelial AP may modulate postischemic kidney fibrosis, depending on the strategy used for AP induction. However, the current study has its limitations. Endothelial autophagy was stimulated by the administration of pharmacologically preconditioned cells. Those may influence the biological behavior of other cell types in the kidney as well. Therefore, we did not use a highly specific strategy for endothelial autophagy activation. Additional experiments are required to analyze the consequences of endothelial autophagy activation in a more selective manner (e.g., transgenic organisms displaying autophagy activation in exclusively endothelial cells). Such studies are on the way. The current investigation was designed as a pilot study intended principally to prove or to exclude postischemic renoprotection after endothelial autophagy activation. By using a transgenic model, functional and structural alterations of the tubular compartment would exclusively occur as a result of modulated endothelial autophagy.

## Conclusion

A renoprotective role for endothelial autophagy in AKI cannot definitely be proposed. Endothelial autophagy may modulate postischemic kidney fibrosis, depending on the strategy used for AP induction.
